# Concept for using magnetic particle imaging for intraoperative margin analysis in breast-conserving surgery

**DOI:** 10.1038/s41598-021-92644-8

**Published:** 2021-06-29

**Authors:** Erica E. Mason, Eli Mattingly, Konstantin Herb, Monika Śliwiak, Sofia Franconi, Clarissa Zimmerman Cooley, Priscilla J. Slanetz, Lawrence L. Wald

**Affiliations:** 1 Department of Radiology, A. A. Martinos Center for Biomedical Imaging, Massachusetts General Hospital, Charlestown, MA 02129 USA; 2 Harvard-MIT Division of Health Sciences and Technology, Cambridge, MA 02139 USA; 3 Department of Physics, ETH Zurich, Zurich, Switzerland; 4 Harvard Medical School, Boston, MA 02115 USA; 5 Department of Radiology, Boston University Medical Center, Boston, MA 02118 USA

**Keywords:** Breast cancer, Medical imaging

## Abstract

Breast-conserving surgery (BCS) is a commonly utilized treatment for early stage breast cancers but has relatively high reexcision rates due to post-surgical identification of positive margins. A fast, specific, sensitive, easy-to-use tool for assessing margins intraoperatively could reduce the need for additional surgeries, and while many techniques have been explored, the clinical need is still unmet. We assess the potential of Magnetic Particle Imaging (MPI) for intraoperative margin assessment in BCS, using a passively or actively tumor-targeted iron oxide agent and two hardware devices: a hand-held Magnetic Particle detector for identifying residual tumor in the breast, and a small-bore MPI scanner for quickly imaging the tumor distribution in the excised specimen. Here, we present both hardware systems and demonstrate proof-of-concept detection and imaging of clinically relevant phantoms.

## Introduction

Breast cancer is the most common cancer in women worldwide. Over two million new cases of breast cancer were diagnosed in 2018, constituting 11.6% of all cancers diagnosed that year (men and women), and 24.2% of all cancers in women^1^. Breast-conserving surgery (BCS, a.k.a. lumpectomy or segmental mastectomy) is the first intervention for the majority (64.5%^2^) of early stage diagnoses, and has been shown to have outcomes similar to mastectomy (complete removal of one or both breasts) when complete tumor resection is achieved^3,4^. However, incomplete resection correlates with local recurrence^5,6^, and secondary surgeries are required in 20–66% of initial lumpectomies^7–12^. Additional surgeries impose risks and challenges including increased costs and complications, as well as cosmetic and emotional burdens on the patient^10,13^.

**Figure 1:**
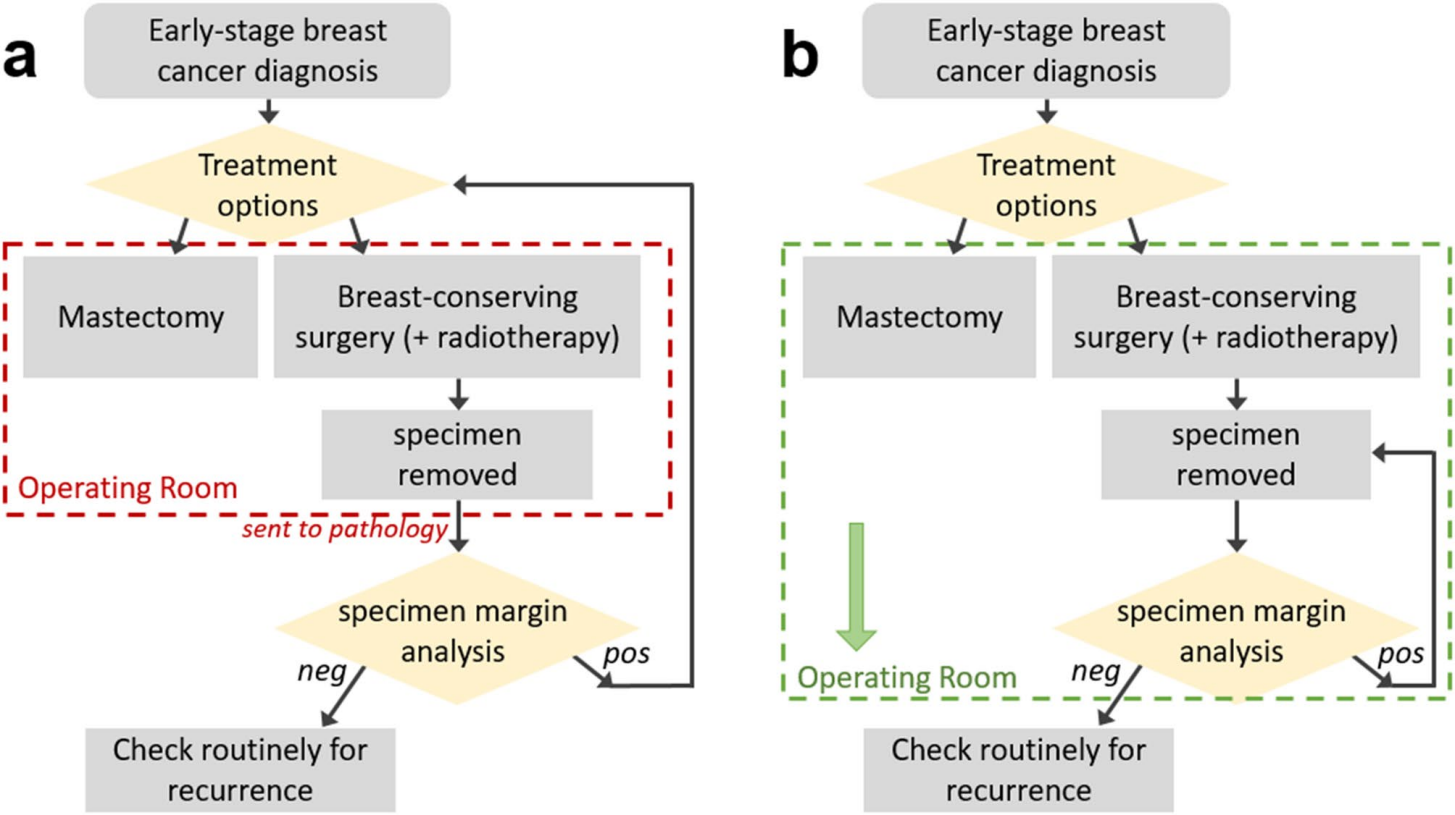
Flowchart of treatment options for early stage breast cancer. **(a)** In current clinical practice, the lumpectomy specimen is removed; after completion of the surgery, the specimen is sent to pathology for analysis. Positive margin results require additional surgeries, either a second lumpectomy or conversion to mastectomy. In contrast, **(b)** shows an improved workflow, in which the specimen margin analysis happens within the operating room, mid-surgery. This would provide the surgeon with immediate feedback, enabling additional tumor removal during a single surgery for a higher likelihood of negative margins.

Local tumor recurrence, a determining factor for reexcision, correlates strongly with margin status^9,14,15^, or the proximity of tumor cells to the inked surface (or “margin”) of the excised tissue. The clinical standard for margin assessment is histopathology, and due to the need for formalin fixation, this assessment is done post-operatively. The need for secondary surgery can be reduced with the use of an *intraoperative* tool that enables the surgeon to iterate tissue removal and margin analysis in real time, enabling greater confidence of clear margins^10,16^. This concept is illustrated in the flowchart in Fig. 1. To gain regular clinical utility, an intraoperative margin assessment technique must provide sensitive and specific information about the margin status, and be fast and easy to use in the operating room.

The problem of positive margins and reexcisions in BCS has been approached with numerous techniques^10,17–23^, addressing both macroscopic tumor localization and microscopic margin assessment. These include frozen section, imprint cytology, cavity shave margins, specimen radiography, micro-CT, radiofrequency spectroscopy, and numerous optical methods, to name just a few. However, as summarized by Maloney et al. (2018), “Technologies to determine margin status have been developed to have high sensitivity and selectivity. However, no current modality has matured into a complete solution to the margin problem currently facing BCS”^18^ due to various limitations, including long analysis times, additional required expertise, and lack of depth penetration.

In this work, we explore a novel approach for margin analysis during BCS: the use of Magnetic Particle Imaging (MPI), in conjunction with an injected superparamagnetic iron oxide nanoparticle (SPIO) tracer. We aim to assess MPI’s potential to be added to the extensive list of tools investigated for this yet unmet need. The potential for SPIOs in breast cancer is highlighted by recent techniques utilizing magnetic nanoparticles and magnetic detectors (Magtrace/Sienna+ and Sentimag, respectively^24,25^) for sentinel lymph node detection and occult lesion localization^20,26^, and as markers to flag specific locations (Magseed^20,27,28^). These technologies use interstitial or intratumoral injections (versus intravenously injected nanoparticles). MPI detection of interstitially injected nanoparticles has also been proposed for use in sentinel lymph localization using a single-sided MPI imager^29,30^. Together, these demonstrations suggest the potential for magnetic nanoparticles in general and MPI in particular for the related task of margin analysis during breast-conserving surgery. Here, we propose the combined use of two novel MPI devices^31–33^ for margin assessment in BCS utilizing an intravenously injected SPIO, and demonstrate their feasibility via clinically relevant phantoms.

### Magnetic Particle Imaging background

MPI is a tracer-based imaging modality first introduced by Gleich and Weizenecker in a seminal *Nature* paper in 2005^34^. MPI utilizes a superparamagnetic iron oxide tracer, identifying and spatially mapping its signature nonlinear magnetization response to an applied AC field. In the presence of the applied drive field (also called “transmit” or “Tx” field) at frequency f$_0$, the SPIO’s nonlinear magnetization includes higher order harmonics of the drive frequency (n*f$_0$, n $=$ 3, 5, 7...). Both the drive field and SPIO response are picked up via Faraday detection in a receive coil, and identification of these higher order harmonics indicates SPIO presence. The drive field must be filtered to suppress harmonics that may be confused for the SPIO signal and f$_0$ must be suppressed in the receive signal to avoid the direct Tx feedthrough. For sufficiently high magnetic field strength, the SPIO magnetization saturates such that the SPIO response is not detectable. This saturation effect is exploited to enable spatial encoding, using magnetic gradient fields (field-free point (FFP) or field-free line (FFL)), and additional AC “shift” fields to move the FFP/FFL for image encoding. Numerous acquisition and encoding schemes have been implemented in preclinical MPI to date^29,34–39^.

MPI is currently undergoing rapid development toward a number of clinical applications, including vascular imaging^40–42^, cell tracking^43,44^, oncology^30,45,46^ including macrophage activity^47^, hyperthermia therapy^48^, traumatic brain imaging^49^, and neuroimaging for stroke and hemodynamic disorders^50,51^. Biomedical applications of MPI have recently been reviewed^52–55^.

MPI and its SPIO tracer have a number of promising features, including zero background signal from tissue and positive contrast (MPI detects only the nonlinear SPIO signal; biological tissue is magnetically linear), high sensitivity (due to the strength of the SPIO magnetic moment, 22 *million* times stronger than the nuclear magnetization detected in high-field MRI), and direct and quantitative tracer detection (in contrast to indirect, qualitative detection as in contrast-enhanced MRI)^56^. As quasistatic magnetic fields are not attenuated in the human body, there are no inherent depth penetration limitations such as those that limit many optical imaging modalities^57^. MPI has also been demonstrated with fast temporal resolution^40^. Finally, SPIOs are stable, have a long shelf life, and are considered safe injected agents with already approved clinical uses^39,58^. These features make MPI promising for addressing the needs of intraoperative breast cancer margin assessment. While the technology is currently preclinical, scaling the hardware to human sizes gives rise to numerous technical challenges^50,59^. We have developed and tested two strategies utilizing small- and medium-sized devices suitable for human clinical intraoperative use.

**Figure 2:**
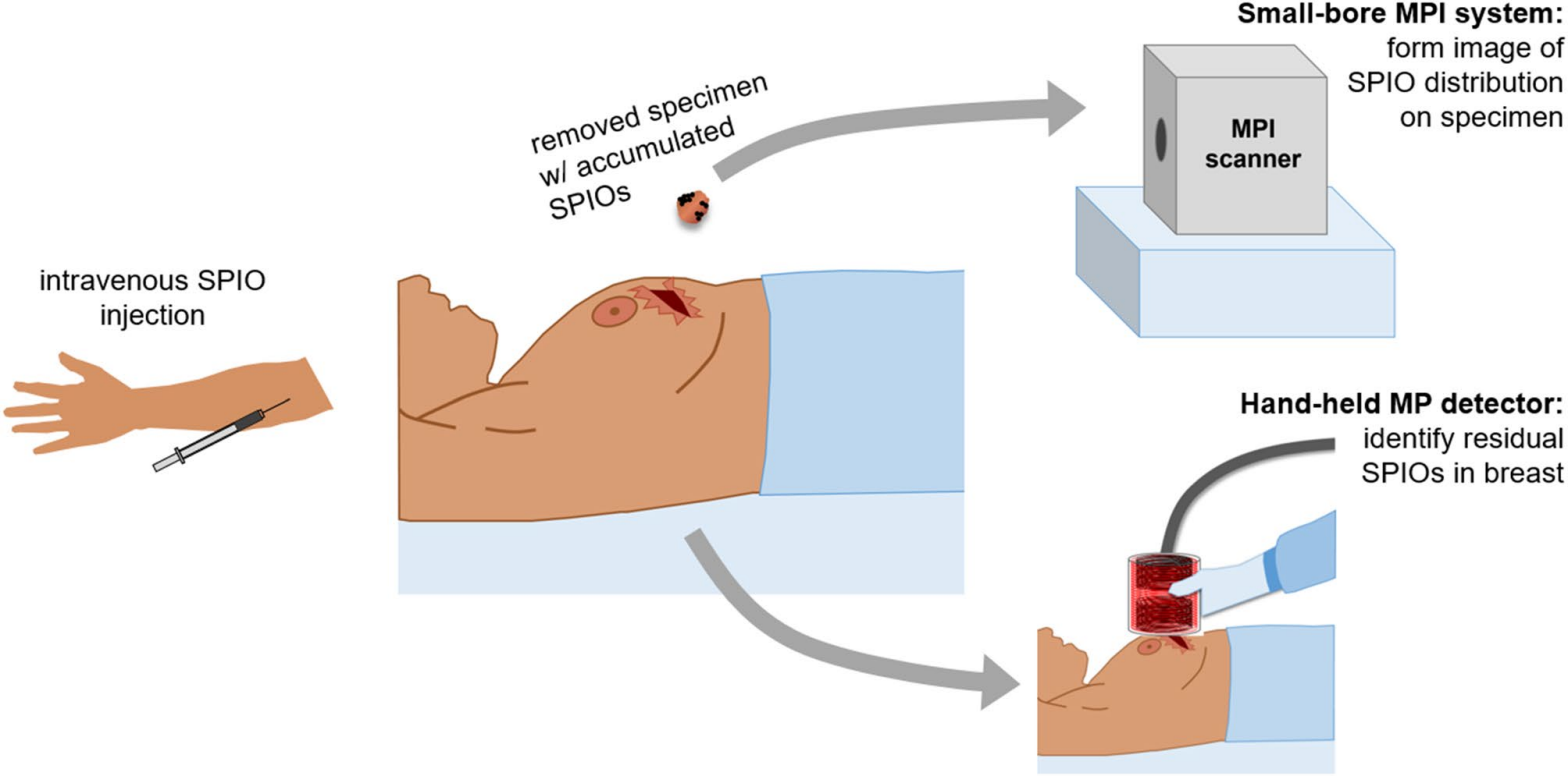
Envisioned MPI workflow during breast-conserving surgery. SPIOs would be injected intravenously prior to surgery with sufficient time for the nanoparticles to accumulate at the tumor and the background signal in the vasculature to be cleared. During surgery, the specimen is removed, and is placed in a small-bore MPI scanner to quickly image the distribution of SPIOs. A hand-held detector would be used at the incision site to detect residual SPIOs still in the breast, indicative of tumor remaining in the breast. Figure created using MS PowerPoint.

### Tumor SPIO accumulation

As MPI detects injected SPIOs only (not the biological tissue itself), SPIOs must serve as tumor markers. Thus, this application relies on high specificity and high sensitivity accumulation. SPIOs are considered safe tracer agents, have routine clinical uses^60–66^, and can be optimized for targeting via size and coating characteristics^61,62^.

SPIOs can accumulate at a tumor via passive mechanisms^67–71^, tumor-associated macrophage uptake^72^, and/or active mechanisms^61,65,73–76^ (use of functional groups or antibodies conjugated to SPIO coating). These phenomena have been widely exploited for targeted tumor therapeutics and imaging. While passive accumulation has been extensively tested in animal models, there is some concern about its reliability for clinical uses in humans^77,78^. Active targeting mechanisms, a potentially high-specificity approach, is an active area of research in the context of breast cancer^61,65,73–76,79^. Du et al. (2019) reviews current clinical application status of both passive and active nanotargeted agents specifically for breast cancer^79^. MPI imaging of passively accumulated^45^ and actively targeted^46^ MPI-tailored SPIO agents have been demonstrated in rodents.

MPI may be considered a two-key problem, requiring (1) hardware with high sensitivity, spatial/temporal resolution, and clinical use feasibility, and (2) safe nanoparticles with application-relevant properties. In this work, we develop the devices needed to take advantage of SPIOs with sufficiently specific tumor uptake and clearance of background SPIOs via the reticuloendothelial system, given the right timing post-injection. Tumor accumulation rates (% injected dose (ID)/g tumor tissue) vary in the literature^80,81^, with a lower bound found in a study using PEG-coated nanoparticles *in vivo* in a xenograft mouse model^81^. Here, total tumor accumulation (calculated as area-under-the-curve), is observed to be 0.3% ID$\cdot$h/g, which we interpret (after accounting for the 2.5 h half-life) to be an accumulation of 0.12% ID/g. However, it is unclear that this value is ultimately translatable to human clinical application. Active targeting has been shown to enable higher accumulation rates than passive targeting^82,83^, and therefore 0.12% ID/g represents a reasonable detection goal.

### Envisioned clinical workflow

Given sufficiently specific SPIO accumulation and sufficient time for background SPIO clearance, MPI can be applied to both the breast at the excision site and to the excised specimen. We envision an MPI BCS workflow as illustrated in Fig. 2. Prior to surgery, the patient receives an intravenous SPIO injection, and sufficient time is allowed for nanoparticle accumulation and local background vascular clearance (the SPIO agent may still remain in other background organs (liver, spleen)^84^, but would sit beyond the hardware’s detection range). During surgery, the specimen is removed, and a hand-held Magnetic Particle (MP) detector can be used at the excision site to identify any residual SPIOs (and thus tumor) in the breast. Simultaneously, the specimen can be inserted into a small-bore MPI scanner to provide a fast image ($\sim$ seconds) of the SPIO distribution in the removed tissue. With information about both the presence of SPIOs (tumor) still in the breast, as well as the spatial distribution of SPIOs (tumor) in the specimen, the surgeon can iterate the process, continuing to remove tissue at indicated sites until achieving a high confidence of full tumor removal.

In this work, we develop and test the two MPI devices. The hand-held MP detector is designed to exploit the principles of MPI in its simplest form. It is a compact, single-sided non-imaging device with drive and receive hardware (no spatial encoding), designed to detect residual SPIO not removed with the initial excision. We design, construct, and show proof-of-concept feasibility of such a device to detect SPIO samples embedded in an anthropomorphic breast phantom.

In a separate device, we test the capabilities of a small-bore imager suitable for placement in the surgical suite to enable quick 3D imaging of the SPIOs within an excised specimen.

## Hand-held MP detector

### Materials and methods

The MP detector is a single-sided device mounted on a flexible arm, such that it can be held and moved around by the surgeon to and from the excision site, as illustrated in Fig. 2. This enables assessment of the presence of detectable levels of tracer. The application assumes that a limited search space around the surgical margin must be covered and that this region is small enough to be exhaustively covered in a timely fashion.

**Figure 3:**
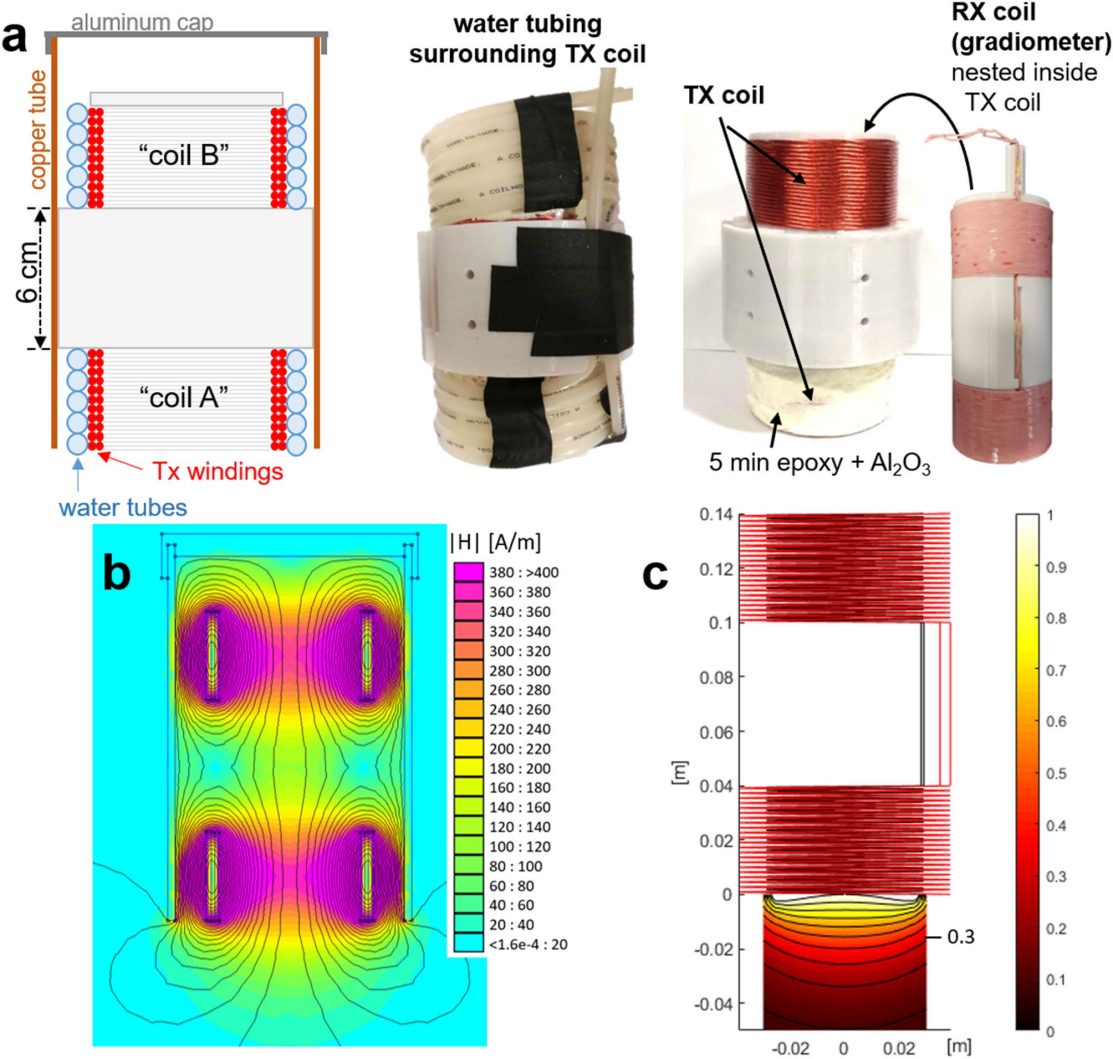
Hand-held MP detector. **(a)** A schematic of the coils and housing is shown on the far left, with coil A and B both wound on a single former, the center section of which extends to the copper tube to which is it mounted. The Tx windings are illustrated in red (as a cross-section), with water tubes wound around them. An aluminum cap shields the back end of the detector. Note the asymmetry of the coil positioning within the copper tube. Figure created using MS PowerPoint. The middle left photo shows the coils wound in water tubing (the black is tape). The middle right photo shows the Tx coil (coil A is covered in the thermally conductive, MPI-inert Al$_2$O$_3$ epoxy mixture, which had not yet been applied to coil B). The far right photo shows the Rx coil (gradiometer). **(b)** Finite element simulation (FEMM 4.2^85^) of the Tx coils in copper tube at 25 kHz to show field-shaping effect of tube. Colormap shows H in A/m per 1 A current. **(c)** Custom simulation of detection sensitivity profile (Tx and Rx coils, no copper tube), using MATLAB (R2018b, https://www.mathworks.com/products/matlab.html). Tx (red coil) simulated with 25 kHz 30 A$_{\mathrm {pk}}$ field, SPIO model based on measured spectrometer data of VivoTrax (Magnetic Insight, Alameda, CA) nanoparticles. Colormap normalized to 1 at center surface of detector (x, y, z) = (0, 0, 0). Iso-contour lines are shown with spacing 0.1, with the 0.3 line indicated.

Single-sided devices have been explored in the field of MPI^29,38,86,87^; for this clinical application, a single-sided, detector-only approach enables a simple, hand-held device. While MPI has no inherent depth attenuation, a single-sided coil has a sensitivity drop-off with distance from the detector, as illustrated in Fig. 3. This feature has the disadvantage that it loses sensitivity for tumors deeper in the breast, but has the advantage of being insensitive to relatively nearby organs with high SPIO content, such as the heart or liver.

Our device is designed for placement outside the incision; it is a larger device mounted on a moveable arm (similar in concept to x-ray devices common in dentist offices). Other single-sided designs could include smaller, pen-like devices (akin to the Sentimag system^24,88^, the MarginProbe device^89^, or an OCT probe^90^).

**Figure 4:**
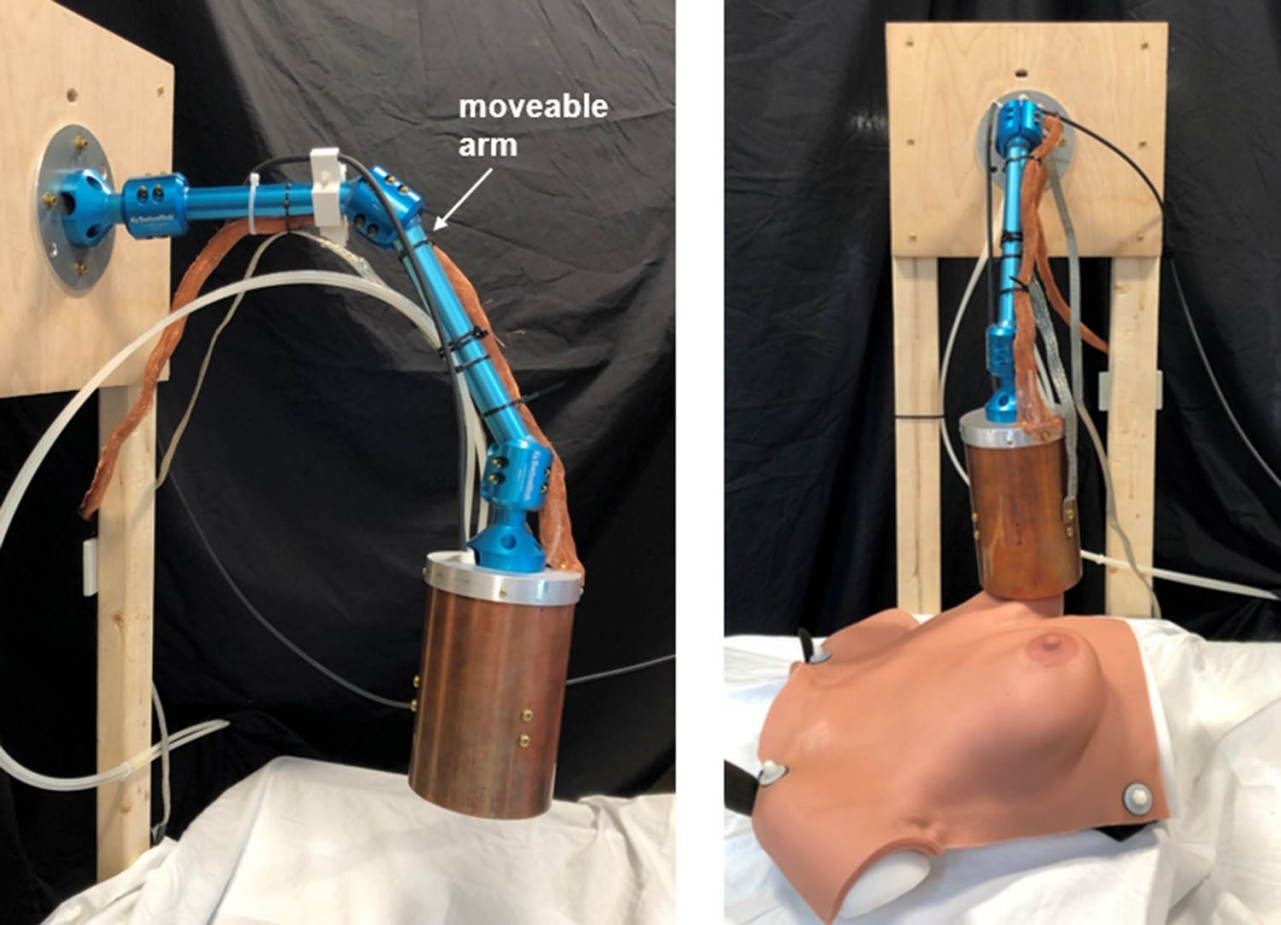
MP detector mounted on moveable arm. Photos at two angles, one with the breast phantom and one without, are shown. The copper tube and aluminum cap are attached to an articulating arm made of anodized aluminum with three joints, enabling a wide range of motion so that the detector can be moved by hand to and from any desired positions. Water tubes and wires are visible and secured to the arm.

The detector consists of two sets of coaxial, solenoidal Tx and receive (Rx) coils, separated by 6 cm, as shown in Fig. 3. The Tx/Rx coil set closer to the detector’s surface picks up SPIO signal, while the second set toward the back end of the detector has an oppositely wound receive coil and serves to cancel the drive field feedthrough. Both coil pairs are epoxied and water-cooled, and the apparatus is housed in a copper tube to confine the stray fields and reduce susceptibility to nearby conductive surfaces as the detector is moved around. Each coil set is 4 cm long; the Tx coil is two 22-turn layers of 16 AWG Litz wire (New England Wire, Lisbon, NH) with an inner diameter of 6.85 cm. The Rx coil, mounted concentrically inside, has two 33-turn layers of 20 AWG Litz with diameter of 5.6 cm. The Tx coils in series have a total inductance of L $=$ 175 H and produce 0.48 mT/A at the surface of the detector. The detector is mounted on an articulating aluminum arm, enabling hand-held movement with many degrees of freedom. Figure 4 shows the mounted detector and an anthropomorphic breast phantom used in experiments (Wearable Breast Self Examination, Anatomy Warehouse, Evanston, IL).

A 25 kHz (drive frequency, f$_0$) signal is produced by an NI USB-6363 DAQ console (National Instruments, Austin, TX), amplified by an AE Techron 7548 power amplifier (Elkhart, IN), and filtered by a custom high-power low-pass filter tuned to f$_0$. The receive chain includes a tuning/notch filter (73 dB attenuation between 3f$_0$ and f$_0$), low-noise preamplifier (Ithaco 1201, DL Instruments, Brooktondale, NY), and bandpass filtering 40–99.9 kHz (SR650, Stanford Research Systems, Sunnyvale, CA). Currently only the 3f$_0$ frequency is recorded with the NI-DAQ console. The drive field is pulsed in short bursts (48–72 ms), with pauses (300–450 ms) between to enable low duty cycle. The beginning of the received pulse can be discarded or the transmit pulse can be multiplied by a ramp function to mitigate transient effects. In either case, this transition period does not fully contribute to signal detection. Each data point recorded represents the 3f$_0$ component of the received signal.

**Figure 5:**
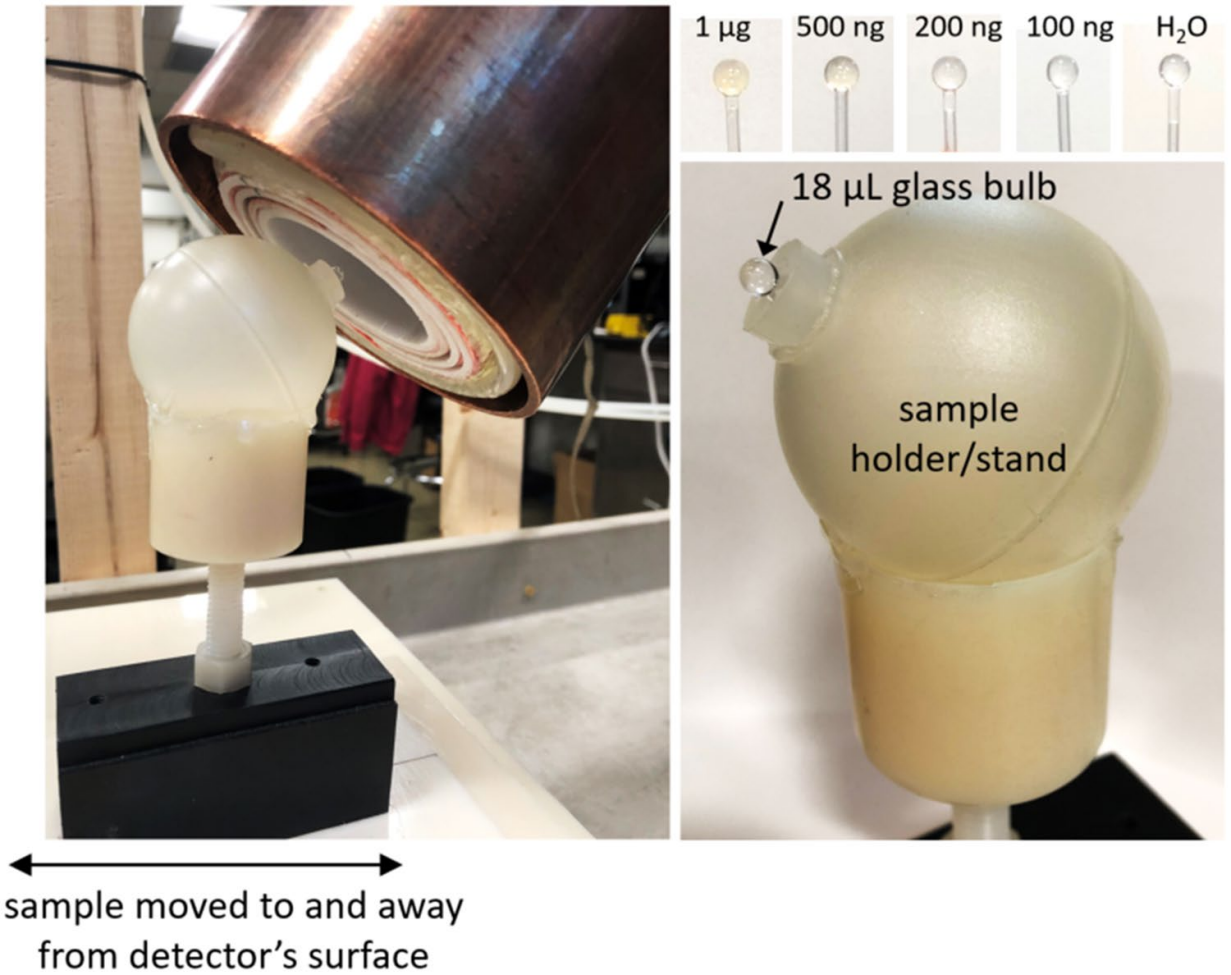
Experimental setup for surface detection. A plastic stand holds the glass bulb SPIO samples, and the detector is positioned such that the samples are approximately at the center of the surface/end plane of the coils. The detector’s mounting arm can be tightened so that it stays in place. The holder can be moved up to and away from this position in a repeatable manner. Samples between 1 µg and 100 ng Fe are tested as well as a control bulb filled only with DI H$_2$O (data shown in Fig. 6).

**Figure 6:**
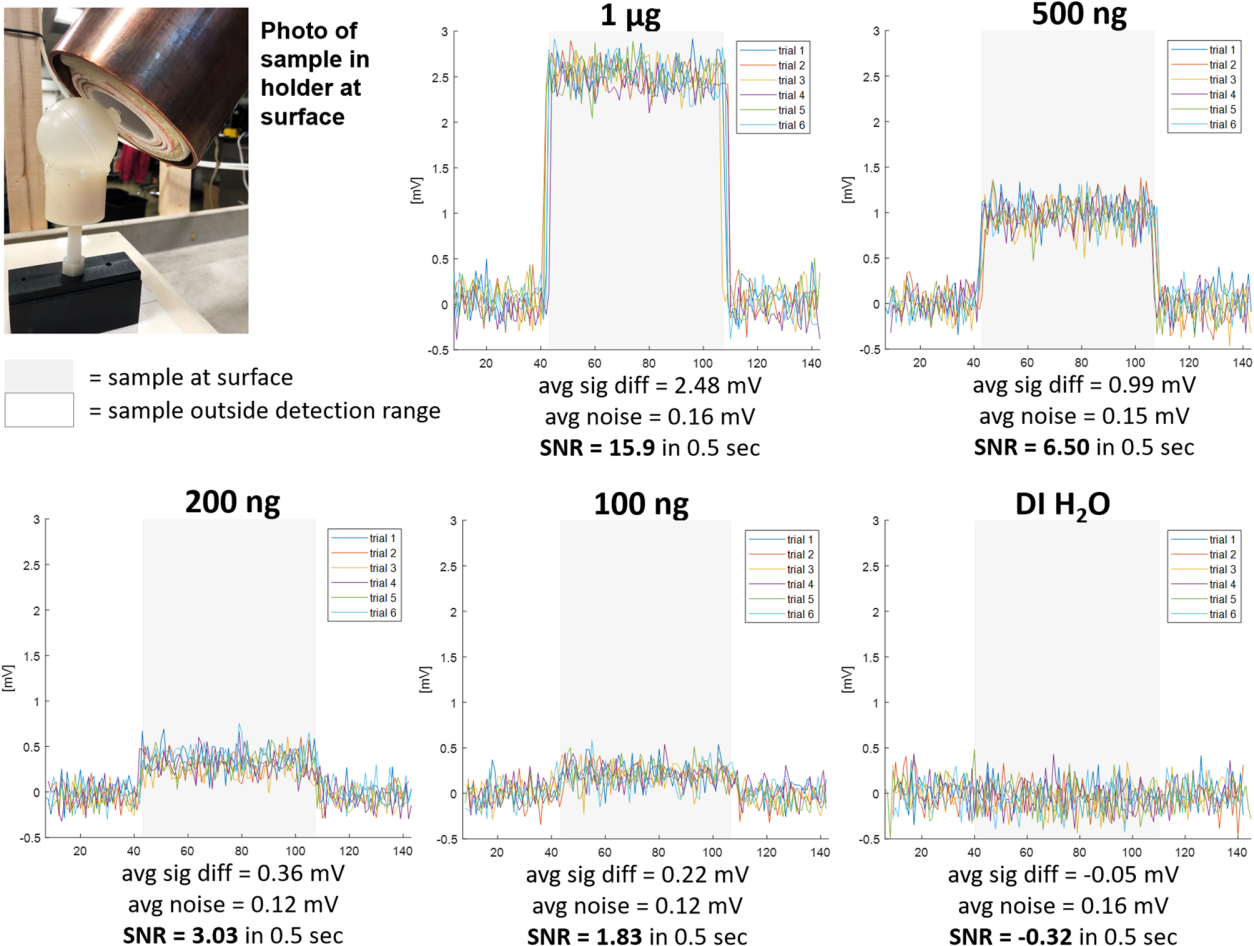
Surface detection results, sample moved. Samples of VivoTrax SPIOs (Magnetic Insight, Alameda, CA) of varying concentrations in 18 µL glass bulbs are moved to (gray shaded region) and away from (not shaded) the surface/end of the detector. The transmit field at the detector’s surface is 25 kHz, 14.3 mT peak, produced with 29.5 A peak current. This is pulsed in 72 ms bursts with a 450 ms pause time, for a 16% duty cycle. The first quarter of the received pulse is discarded to remove transient effects, for a total 54 ms received pulse every 450 ms, and each data point shows the magnitude of the 3f$_0$ frequency component of the received pulse. Preamplifier gain is 500. In post-processing, a two-term exponential magnitude drift is removed in order to overlay the trials. DI water in the same glass bulb and plastic sample holder serves as an experimental control. A plot of the measured signal magnitudes (“avg sig diff”) as a function of SPIO quantity can be found in supplementary figure S4 to illustrate the linearity of MPI signal with iron content.

**Figure 7:**
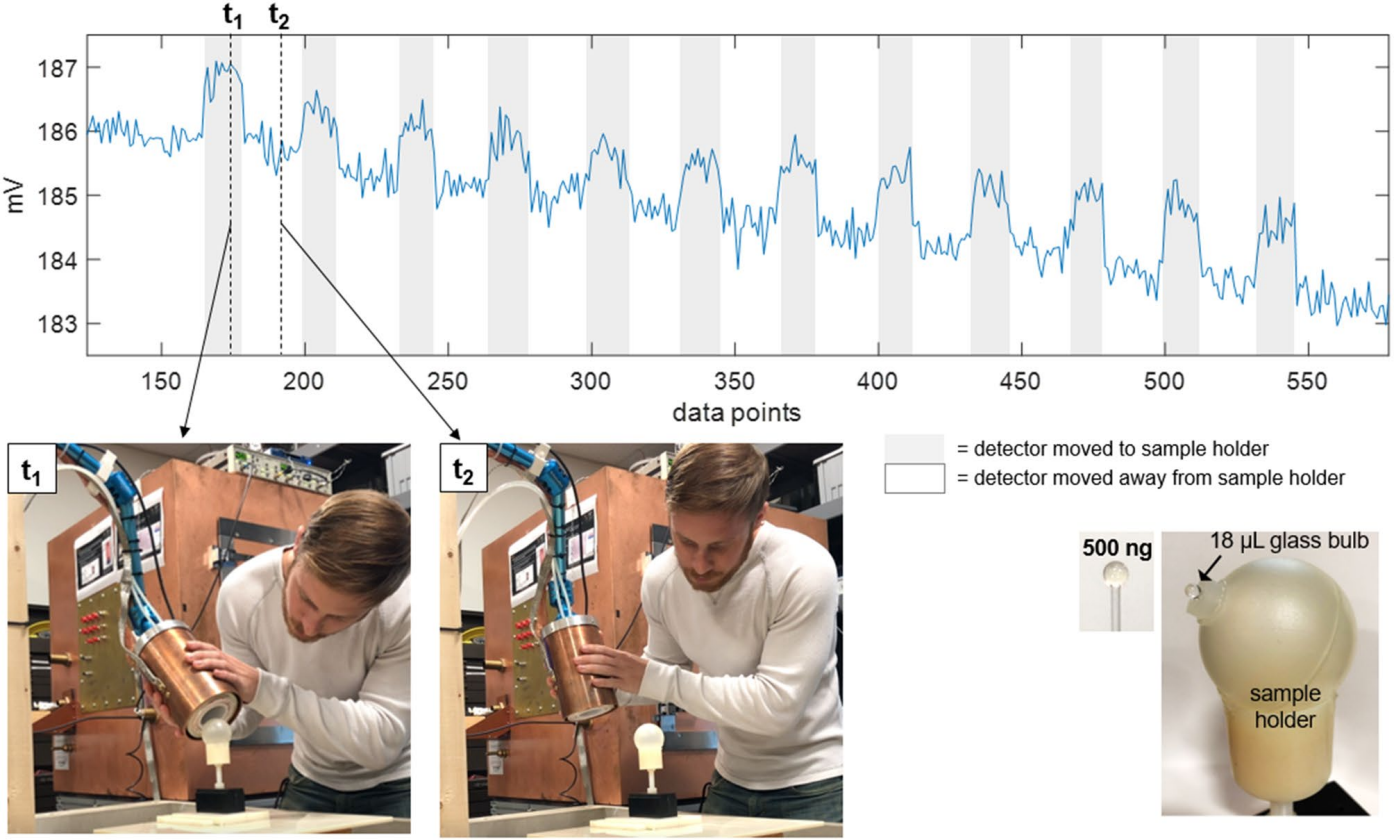
Surface detection results, detector moved. Detection of a 500 ng Fe SPIO sample, for which the sample is stationary and the detector is moved by hand to and away from it. Shaded gray regions indicate when the detector is moved such that the sample is near its surface. A slow drift of a few mV in the baseline signal is apparent over the 2-min. time-series. For two time points, t$_1$ and t$_2$, still frames from a video of the experiment are shown. (See supplementary material S1 for video.) Each data point is the magnitude at 3f$_0$ of the FFT of a 36 ms long time trace acquired during the application of a 48 ms long drive pulse. A 300 ms pause follows each 48 ms drive pulse. Tx amplitude is 14.3 mT peak, produced with 29.5 A peak current. Rx chain includes Ithaco 1201 preamp with G $=$ 500 and SR650 band-pass filtering 40–99.9 kHz. No post-processing used.

We make the assumption that SPIO can accumulate at the tumor with a rate of 0.12% ID/g tumor tissue (see “Tumor SPIO accumulation” section above) and that background SPIOs will be cleared, such that there is no SPIO signal from healthy tissue. Based on this assumption, a typical SPIO dose of 5 mg/kg Fe, and a 65 kg patient with a 1.35 mm diameter tumor with a density of 1 g/cm$^3$ (that of water), the hand-held device must be capable of detecting 500 ng Fe. Detection of 200 ng Fe is needed to reveal a 1.0 mm diameter volume of residual tumor.

We present three proof-of-concept experiments. First is surface detection of varying concentrations of SPIOs (VivoTrax, Magnetic Insight, Alameda, CA) in 18 µL glass bulbs, containing 1 µg down to 100 ng Fe, moved to and away from the detector’s surface. A 0 ng Fe (DI H$_2$O) control bulb ensures no false detection due to coil loading. Figure 5 shows the experimental setup. The raw acquired data has a baseline drift; to overlay the trials, a two-term exponential fit is subtracted from the time-series data in post-processing. Signal-to-noise ratio (SNR) is calculated as the mean of the difference in signal when the sample is at the detector’s surface vs. pulled away, divided by the standard deviation of the signal when the detector is pulled away.

The second experiment more directly mimicked the clinical setting, testing hand-held use in which the SPIO sample is stationary and the detector is moved to and away from it. Video of the experiment (as well as screen recording of the data control software) was acquired during the experiment. This video is provided in supplementary material S1.

**Figure 8:**
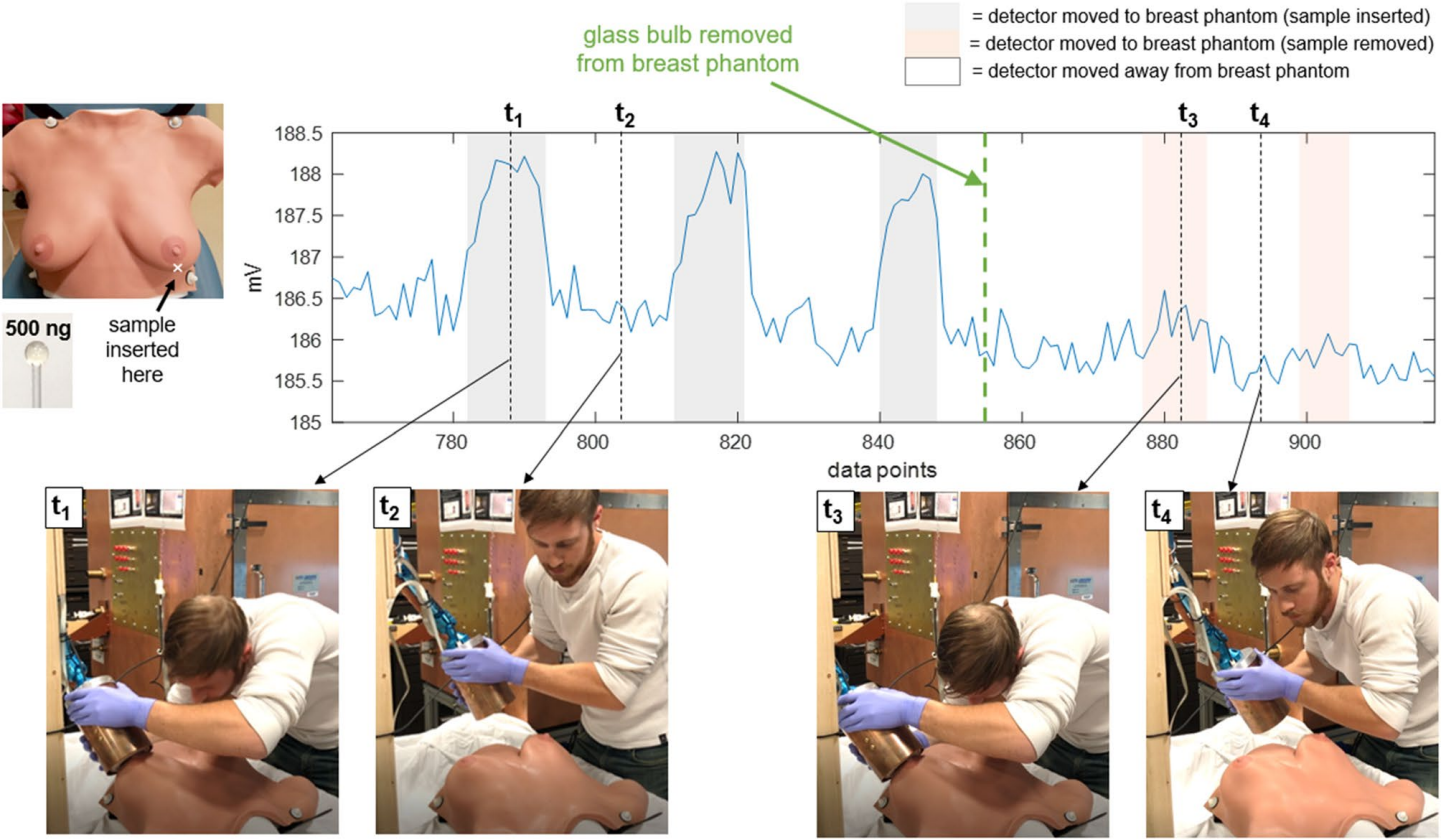
Breast phantom detection results, detector moved, left breast lower outer quadrant. Detection of a 500 ng Fe SPIO sample embedded in the anthropomorphic breast phantom in the lower outer quadrant of the left breast. The detector is held by hand and moved around during data acquisition to and away from the breast at the sample’s location. Four time points (t$_1$–t$_4$) are selected to show still frames from a video of the experiment. (See S2 for video.) The SPIO sample (in glass bulb) is removed from the breast phantom at data point 855 (thick green dashed line). The acquisition scheme, receive chain setup, and data analysis are the same as described in the caption of Fig. 7.

**Figure 9:**
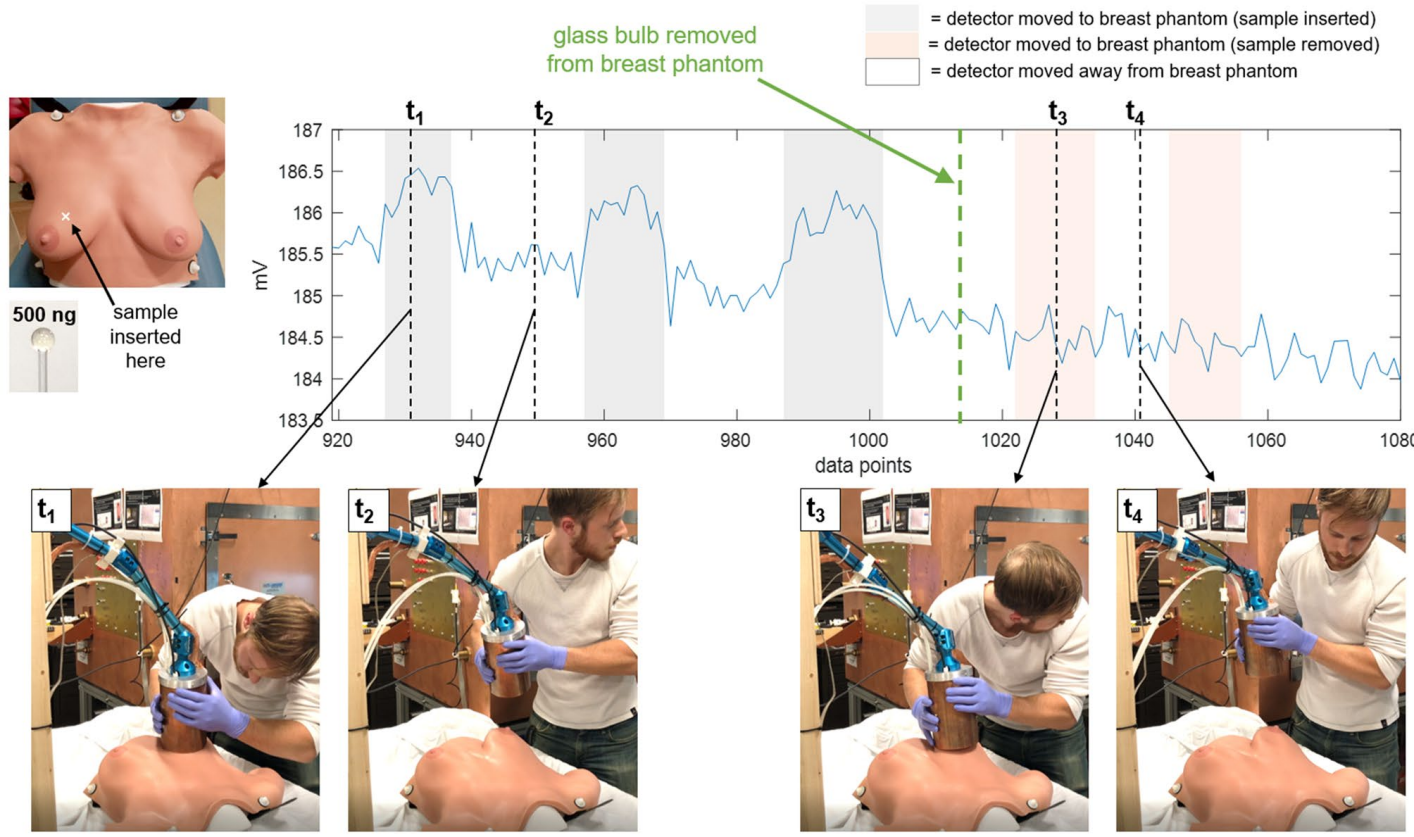
Breast phantom detection results, detector moved, right breast upper inner quadrant. Detection of a 500 ng Fe SPIO sample embedded in the anthropomorphic breast phantom in the upper inner quadrant of the right breast. The detector is held by hand and moved around during data acquisition to and away from the breast at the sample’s location. Four time points (t$_1$–t$_4$) are selected to show still frames from a video of the experiment. (See S3 for video.) The SPIO sample (in glass bulb) is removed from the breast phantom at data point 1016 (thick green dashed line). The acquisition scheme, receive chain setup, and data analysis are the same as described in the caption of Fig. 7.

For the third experiment, the SPIO samples (in glass bulbs) are embedded in the anthropomorphic breast phantom, and detection is demonstrated by holding the detector and moving it to and away from the breast phantom at the known locations of the samples. The samples (each 500 ng VivoTrax) were placed in two locations (one in the lower outer quadrant of the left breast, one in the upper inner quadrant of the right breast), to vary both the material (gel/rubber vs. hard plastic) and surface curvature of the breast phantom, as these could affect coil loading and positioning. Finally, to verify the detection, the samples are removed and detection tested moving the detector to the same locations. Video and screen recording of data control software was acquired during the experimental procedure. These videos are available in supplementary materials S2 (sample in left lower outer quadrant location) and S3 (sample in right upper inner quadrant location). The person demonstrating hand-held use of the detector in Figs. 7, 8, and 9 and supplementary videos S1, S2, and S3 is E. Mattingly, second author. The person briefly in frame in supplementary videos S2 and S3 to remove the samples from the anthropomorphic breast phantom is E. E. Mason, first author. No human subjects, animals, or biological tissue samples were used in this work.

### Results

Figure 6 shows the SPIO signal level with the sample placed at the detector’s surface and distant. The system is sensitive to 100 ng Fe with SNR 1.83 using a pulsed Tx signal of 72 ms and a 450 ms pause time. The 0 ng Fe phantom is not detectable (SNR < 1). The maximum depth at which the instrument can detect a 500 ng sample with SNR $=$ 2 can be inferred from the contour plots in Fig. 3c. Given that Fig. 6 shows detection of 500 ng at the surface of the detector with SNR $=$ 6.5, we estimate that the maximum depth for 500 ng will be at approximately the 0.3 contour of Fig. 3c. This corresponds to a depth of $\sim$2.5 cm from the center of the coil’s surface.

Figure 7 shows results from the second experiment, in which the detector is moved by hand to and away from the stationary phantoms. Still frames from the video (see supplementary material S1) are included for two time points, t$_1$, when the detector is held up to the sample, and t$_2$, with the detector pulled away. The baseline drift can be seen in the time-series data.

Figures 8 and 9 show detection of samples embedded in the anthropomorphic breast phantom in two locations (full videos available in supplementary materials S2 and S3, respectively). For the lower outer left breast location, when the sample is removed and the detector moved to that location, small perturbations in the baseline can be seen, indicating some coil loading or parasitic effects due to the proximity of (and/or physical contact with) the breast phantom (this can be seen in the orange shaded regions). For the upper inner right breast location, no baseline perturbations are detectable.

### Discussion

The detector is capable of detecting an estimated clinically relevant quantity of SPIO (100 ng Fe VivoTrax). Assuming tumor SPIO accumulation of 0.12% ID/g, the 100 ng Fe sample represents the expected accumulation in a 790 µm diameter tumor. The detector’s feasibility for hand-held intraoperative use is strongly supported by the experiments in which the detector is moved to and away from stationary samples (either in the plastic holder or the anthropomorphic phantom). The detector is held and moved with minimal compromising changes in the baseline signal and with no addition of noise, and it detects a 500 ng Fe sample embedded in different areas of the breast phantom with high sensitivity.

For one of the two sample locations in the breast phantom, a small baseline elevation is present when the sample is removed. Such false baseline perturbations, due to body-loading or parasitic effects, present a critical challenge for the detector’s functionality, as the detector has no ability to distinguish an elevated baseline from a true SPIO signal with its current acquisition and data processing paradigm. Additionally, we note a slow baseline drift. This is likely due to thermal effects, either in the Tx coil (thermal contact between the water tubing and the Tx coil has room for improvement), and/or in the capacitors of the Tx filter (these can have active temperature control in future developments). Baseline drift is problematic when it is fast compared to the rate at which the detector is moved to and away; for this application, the drift is relatively slow and so does not inhibit detection, and is shown here without post-processing such as baseline drift removal. For a clinical implementation, it would be beneficial to mitigate this effect as indicated above in hardware and/or in digital signal processing to further simplify data presentation to the clinician.

We also note that here, voltage amplitude was used as the metric for tracer detection; a more meaningful metric could be used in a clinical device, such as report of SNR or a statistical metric of expected false-positive confidence level. Overall, the results presented demonstrate that a single-sided hand-held MPI detector has the potential for use as a fast intraoperative device, with the sensitivity and stability for detection of small quantities of residual tumor.

## Lumpectomy specimen imaging with small-bore scanner

### Materials and methods

The MPI scanner for intraoperative specimen imaging is envisioned as a compact and portable tabletop or cart-mounted system that can be placed in the operating room and produce fast 3D images of the spatial distribution of SPIOs in the excised tissue. We test a small-bore, mechanically rotated 2D field-free line (FFL) permanent magnet-based imager for this purpose. The imager was previously described^31,32^, and design details and specifications are available open source at https://os-mpi.github.io/^33^.

**Figure 10:**
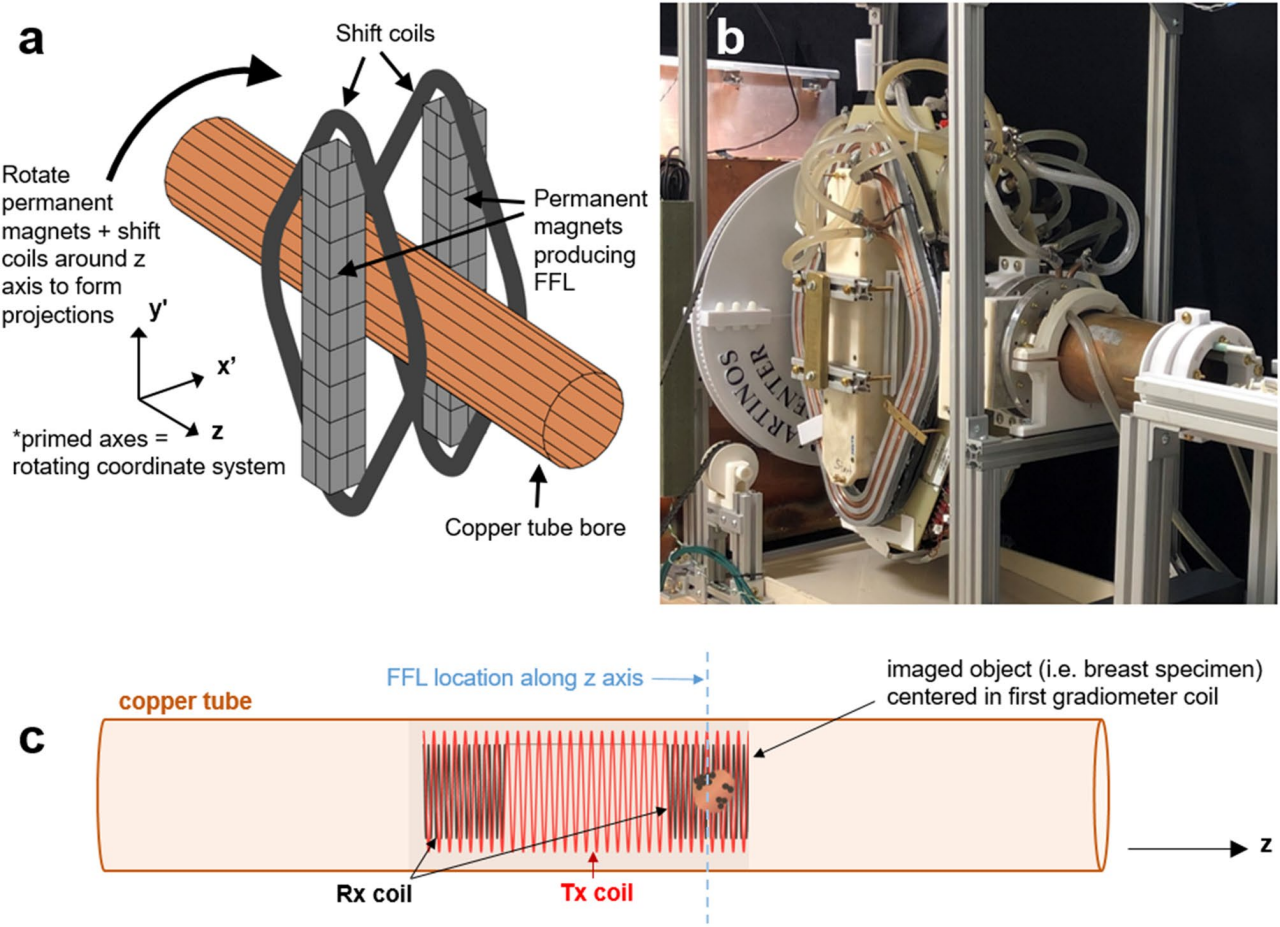
Small-bore imager. **(a)** Schematic of imager, showing the permanent and electromagnet hardware (permanent magnets and shift coils) which rotates about the copper tube bore with the illustrated $(x', y', z)$ coordinate system. Figure made using MATLAB (R2018b, https://www.mathworks.com/products/matlab.html). **(b)** Photo of the imager. The permanent magnets are in an ABS plastic housing, the diamond-shaped shift coils are covered by copper tubing for water cooling (fitted in a milled aluminum plate), and the large gear (3D printed plastic) used to rotate the hardware can be seen behind them. **(c)** The Tx coil (red) and gradiometer Rx coil (black) are illustrated within the copper tube; the tube axis is along *z*. A dashed blue line indicates the imaging plane, i.e., the location of the FFL. The object to be imaged (excised breast specimen), is positioned in the first of the two gradiometer coils. Figure created with MS PowerPoint and MATLAB (R2018b, https://www.mathworks.com/products/matlab.html).

A schematic and photo of the imager are shown in Fig. 10a and b, respectively. Rare earth NdFeB permanent magnets (PMs) produce the FFL along $y'$ (primed axes referring to a mechanically-rotating coordinate system) and electromagnet (EM) shift coils (diamond-shaped, circumscribing the PMs) sweep the FFL across the projection axis, $x'$. The FFL is produced by permanent magnets (two 2” x 2” x 16” N48), and has a measured gradient strength of 2.83 T/m. The EM shift coils are each 250 turns of 14 AWG magnet wire, water-cooled, and produce a 1.31 mT/A field. A 30 A peak current sweeps the FFL across a $\sim$3 cm field of view (FOV).

A copper tube bore is stationary in the (*x*, *y*, *z*) frame and houses the transmit and receive coils. The PM and EM hardware is mechanically rotated about the bore by a motor to acquire 1D projections at multiple angles, using slip rings to enable continuous rotation (rather than having to change rotation directions to avoid twisting wires). The transmit/receive coils and signal chain are similar to that of the MP detector, with a bore diameter of 5 cm. The drive coil is a 12 cm long solenoid with two 41-turn layers of 16 AWG Litz wire (New England Wire). It has L $=$ 195 µH and produces 0.62 mT/A at the center of the imaging FOV (2 cm from end of coil). A gradiometer receive coil is epoxied concentrically inside the drive coil. Its two oppositely wound 20 AWG Litz wire coils (4 cm long, 66 turns each, 4 cm separation) provide first-order drive cancellation. The signal chain utilizes a NI DAQ console and a drive frequency of f$_0$ $=$ 25 kHz. This signal is amplified by an AE Techron 7548 power amplifier, and filtered by a custom high-power low-pass filter tuned to f$_0$. On the receive side, a custom tuning/notch filter provides a measured 60 dB attenuation between 3f$_0$ and f$_0$, followed by further low-noise amplification (Ametek Signal Recovery 5113, Scientific Instruments, Berwyn, PA) and bandpass filtering (SR650, Stanford Research Systems).

A typical image acquisition sweeps the FFL with either a triangular or sinusoidal shift waveform and acquires 27 projections covering 180$^\circ$ with 66 readouts per projection and 150 cycles of the 25 kHz transmit field per readout. The projections are then reconstructed to form a 2D image utilizing a model-based preconditioned conjugate gradients minimization; this method uses a numerical model of the FFL smeared by a Gaussian (empirically determined $\sigma$ = 1.5 mm), which is swept and rotated according to the measured shift current and rotation angle. This accounts for the sensitivity width as well as imperfections in shift current and angular position, which are not represented in a simple iradon transform. 3D imaging can be enabled by mechanically translating the object (e.g. excised specimen) along the bore (*z* axis). Figure 10c illustrates the transmit and receive coils positioned inside the copper tube, with the breast specimen positioned inside the first half of the gradiometer receive coil (the *z* location of the FFL and location of imaging slice).

The imager can produce 2D images in down to 2.9 s; this temporal resolution is not a lower bound, as the system can rotate and image faster than this. However, image speed comes at the expense of SNR and further temporal resolution improvement is unnecessary for this intraoperative application. Spatial resolution in MPI is dependent on SPIO properties and gradient strength; this imager’s spatial resolution has been estimated to be $\sim$2–3 mm for VivoTrax. The imager is sensitive to 100 ng Fe (VivoTrax) (in a 3 mm bulb) in a 5 s image with SNR $=$ 5.

**Figure 11:**
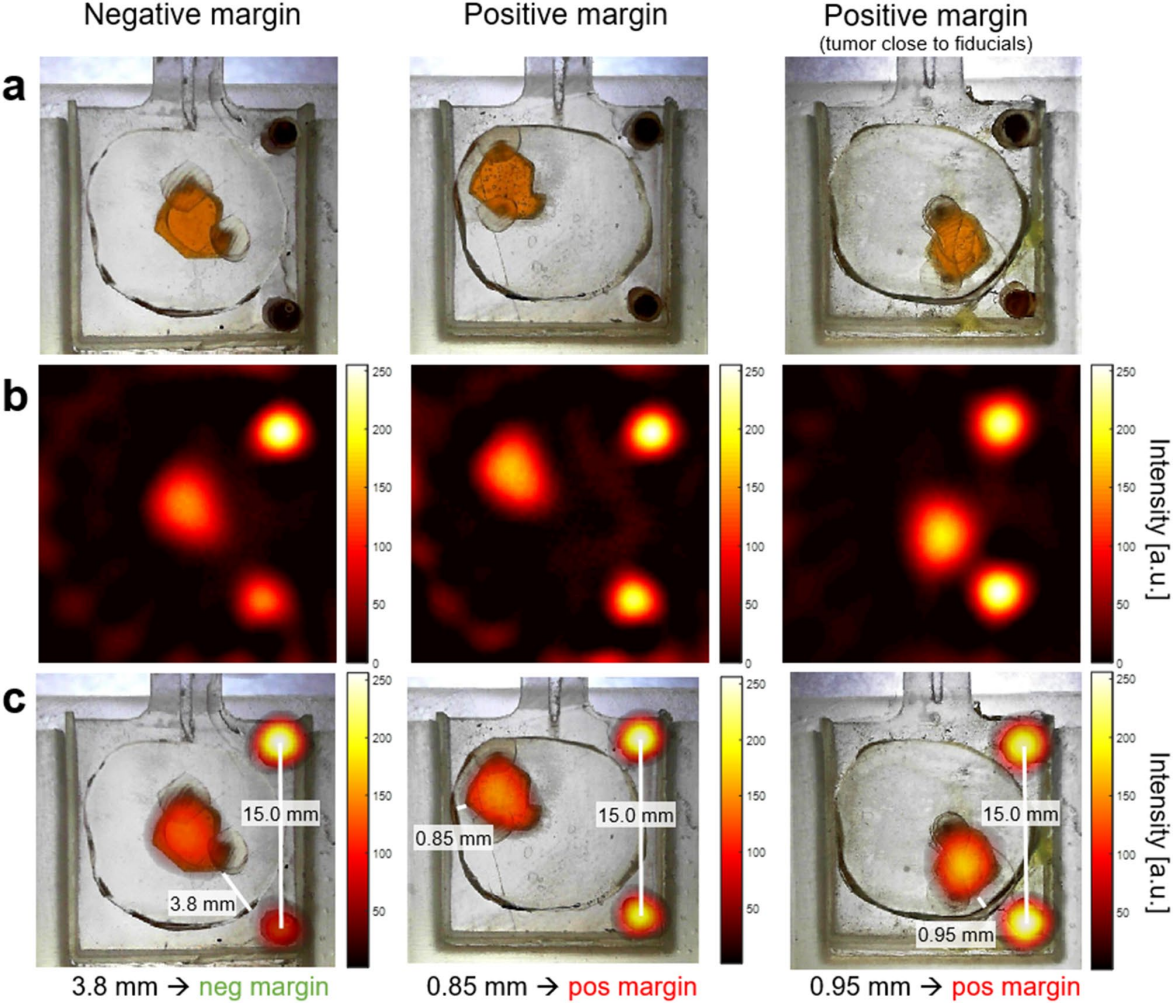
Lumpectomy specimen phantoms, MPI images, and co-registration. **(a)** Optical images of lumpectomy specimen phantoms. The “tumor” is a cavity with a maximum diameter of $\sim$6.5 mm filled with 0.5 mg/mL VivoTrax (51.2 µg total Fe quantity). The fiducials are 1.75 mm diameter cylinders filled with undiluted (5.5 mg/mL) VivoTrax (61.1 µg total Fe quantity). The “healthy tissue” is 3D print material containing no SPIOs. Negative margin is defined as tumor > 1 mm from specimen’s surface; positive margin is defined as tumor $\le$ 1 mm from surface. **(b)** Each MPI image is acquired in 10.7 s using a triangular-waveform shift field with 27 projections, 66 readouts per projection, and 150 Tx cycles per readout. Receive chain has total G $=$ 1000. Image reconstructed with model-based preconditioned conjugate gradient recon. Each MPI image is scaled to its individual maximum. **(c)** MPI images from (**b**) are co-registered with the optical images of the phantoms from (**a**) using the fiducial locations. The distances between the tumor edge and the specimen margin are measured (using the Distance Tool in MATLAB (R2018b, https://www.mathworks.com/products/matlab.html)), correctly classifying the specimen phantoms as negative or positive.

We developed and 3D printed “lumpectomy specimen” phantoms, in which the healthy tissue of the excised specimen is the 3D printed plastic (no SPIOs), and the “tumor” is a cavity filled with a chosen SPIO concentration relevant for passive accumulation rates. Based on specimen and tumor sizes found in the literature^91–94^ and the imager’s $\sim$3 cm field of view, the phantoms are designed with specimen diameter of $\sim$1.6 cm and tumor diameter $\sim$6.5 mm, with two 2.5 mm fiducials. The phantom is a 5 mm-thick 2D slice for simplicity and proof-of-concept. The arbitrarily shaped “tumor” is filled with a 0.5 mg/mL Fe solution for a total of 51.2 µg Fe. Assuming a 5 mg/kg dose and 65 kg patient, this corresponds to tumor accumulation of 0.154% ID/g. The fiducials are filled with undiluted VivoTrax and each contains 61.1 µg Fe. A margin is considered positive/close if the tumor is $\le$ 1 mm from the inked surface, and negative if > 1 mm from the surface^92,93^. Three versions of the lumpectomy specimen phantoms are developed: a negative margin in which the tumor is centered and $\ge$ 3.8 mm from the margin, and two positive margin phantoms in which the tumor is as close to the edge as feasible with 3D printing ($\sim$0.8–0.9 mm), one of which has the tumor far from the fiducials and the other close to the fiducials to ensure the fiducial-tumor proximity does not produce artifacts. Photos of the three phantoms are shown in Fig. 11a.

### Results

Figure 11b shows MPI images of the three “lumpectomy specimen” phantoms, each acquired in 10.7 s. In Fig. 11c, these are shown overlaid onto the optical images of the phantoms using the fiducial locations. By measuring the distance between the tumor edge and the specimen margin, all three images are classified correctly to their respective margin types (positive/negative) and provide visual information for the location of the tumor within the specimen.

### Discussion

Imaging of phantoms representative of excised breast specimens is demonstrated with high sensitivity and with 10.7 s imaging times, fast enough for intraoperative application, and fiducial coregistration is utilized to show tumor location and proximity to margins. The differing intensities between the fiducials is most likely due to imperfect fiducial shape/filling/sealing of the 3D printed wells in these phantoms, since the 3D printer does not always print repeatable small cylindrical wells. Also, the lower fiducial in the negative margin phantom appears to have a small air bubble. Even with this MPI scanner with spatial resolution $\sim$2–3 mm, points closer can still be distinguished given sufficient SNR by locating the centers of their overlapping point spread functions. Ultimately, increased gradient strength will be desired to improve spatial resolution for clinical application.

To be useful in a clinical setting, the MPI must be extended to imaging a full 3D volume. With a 2D FFL scanner like the one used here, this can be done by imaging multiple 2D planes as the sample is moved through the bore by a motorized bed. In this process, out-of-slice imaging artifacts must be addressed by either a 3D model-based reconstruction or further encoding along the bore. These approaches have been demonstrated in x-space^36,95^ or system matrix^96^ reconstructions. To assess the degree to which the detected nanoparticles near the surface of the specimen, it is important to have 3D knowledge of the surface location. Since disease-free tissue is free of nanoparticles and thus invisible to MPI, this requires a second co-registered imaging modality. Since only the surface location is needed, this could be readily supplied by a 3D optical surface scanner^97,98^ prior to insertion in the MPI. The results in Fig. 11 show this registered with 2D optical images as opposed to 3D. Alternatively, the surface information could be provided by a combined MPI/CT^99^ or MPI/MRI scanner^100^.

## Conclusion

For the clinical need of improved intraoperative margin assessment in BCS, we propose a two-part MPI solution involving a hand-held MP detector and a small-bore MPI imager, used along with an injected SPIO agent as a tumor marker. MPI offers high sensitivity detection and imaging with fast acquisition times, and can both detect residual SPIO in the excision cavity as well as image its spatial distribution on the removed specimen. With these features, MPI can be used iteratively during surgery to better achieve complete tumor removal and thus a higher likelihood of negative margins. Both proposed hardware systems are designed, constructed and validated in phantoms.

The hand-held detector is demonstrated to have clinically relevant sensitivity (down to 100 ng Fe, representing a 790 µm diameter tumor), as well as stable and reproducible hand-held use for samples embedded in an anthropomorphic breast phantom. This demonstrates its ability to detect small volumes of residual tumor tissue in the breast after initial specimen excision.

A small-bore 2D FFL projection imager is developed with a 5 cm imaging bore, 2.83 T/m gradient field, and 3 s temporal resolution with the ability to image continuously. The imager is sensitive to 100 ng Fe (VivoTrax) (in a 3 mm bulb) in 5 s with SNR of 5, and has spatial resolution $\sim$2–3 mm. We demonstrate imaging of phantoms representative of excised breast specimens, showing tumor location and proximity to margins with high sensitivity and with 10.7 s imaging times, fast enough for intraoperative use. This serves as a proof-of-concept demonstration of MPI’s potential for fast imaging of excised specimens with relevant tracer levels. Combined with a 3D optical surface scan or other co-registered structural imaging modality, small-bore MPI may be able to determine the distance between tumor and specimen surface and thus provide a timely margin assessment to the surgeon. Together with the hand-held detector, this work demonstrates the potential for MPI as a clinically realizable solution to the problem of positive margins in breast-conserving surgery.

## Supplementary Information

Supplementary Information.

Supplementary Information.

Supplementary Information.

Supplementary Information.

Supplementary Information.
