# Supplementary figures and images for: Concept for using magnetic particle imaging for intraoperative margin analysis in breast-conserving surgery
Source: Sci Rep. 2021 Jun 29;11:13456. doi: 10.1038/s41598-021-92644-8 (PMC8242088; doi:10.1038/s41598-021-92644-8)

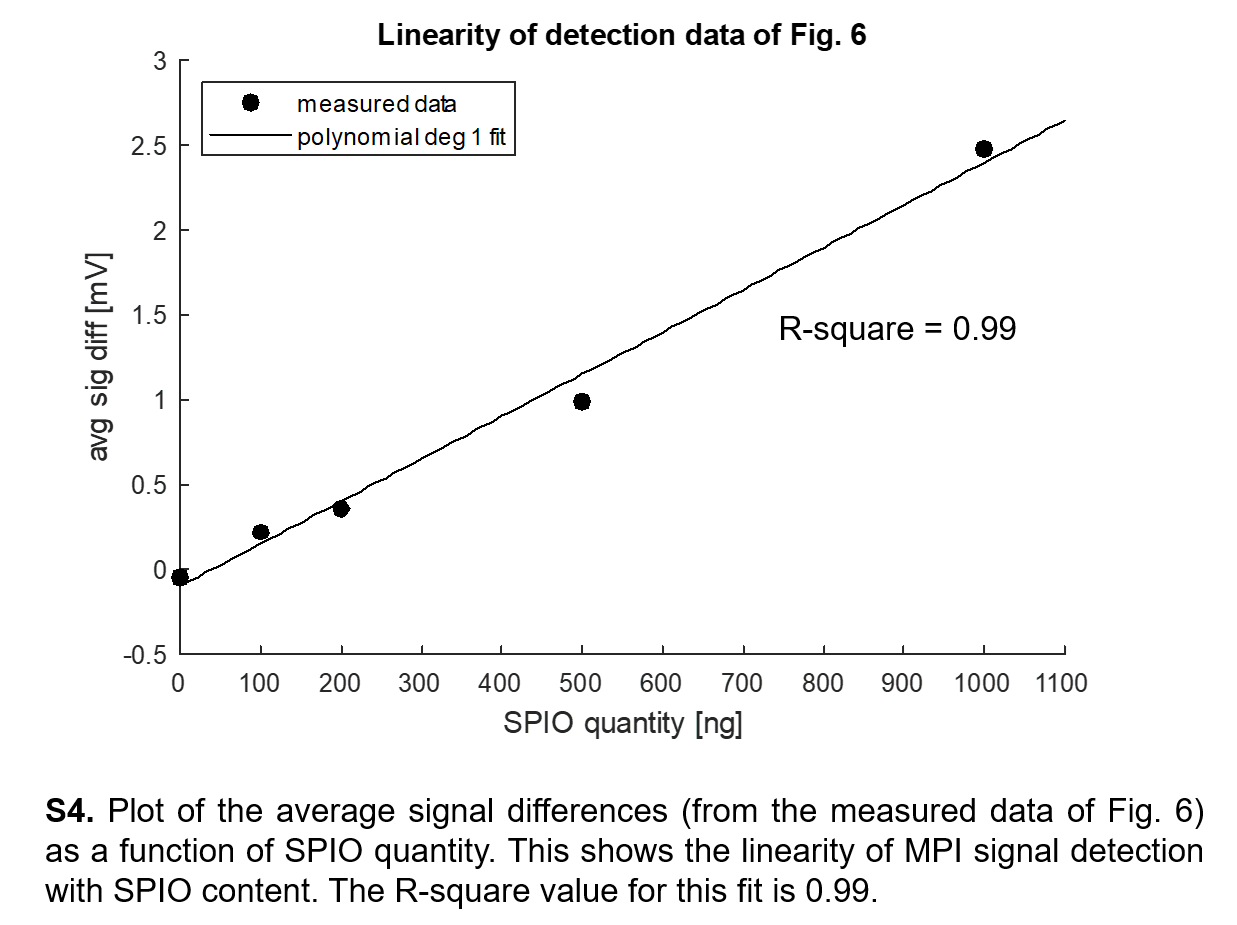

Supplement: Supplementary file 4 — Supplementary Information. [file 41598_2021_92644_MOESM4_ESM.png]
