# Supplementary material for: Concept for using magnetic particle imaging for intraoperative margin analysis in breast-conserving surgery
Source: Sci Rep. 2021 Jun 29;11:13456. doi: 10.1038/s41598-021-92644-8 (PMC8242088; doi:10.1038/s41598-021-92644-8)
Supplement: Supplementary file 5 — Supplementary Information. [file 41598_2021_92644_MOESM5_ESM.pdf]

Supplementary Videos S1, S2, S3 and Supplementary Figure S4

Manuscript Title:

Concept for using Magnetic Particle Imaging for intraoperative margin analysis in breast-conserving surgery

Author list:
Erica E. Mason, Eli Mattingly, Konstantin Herb, Monika Śliwiak, Sofia Franconi, Clarissa Zimmerman Cooley, Priscilla J. Slanetz, Lawrence L. Wald

S1. Video of experiment displayed in Fig. 7, showing the video recording alongside the screen recording of the real-time data acquisition. The detector is moved by hand to and away from a stationary 500 ng Fe SPIO phantom.

S2. Video of experiment displayed in Fig. 8, showing the video recording alongside the screen recording of real-time data acquisition. An SPIO sample (500 ng Fe in a 3 mm-diam. glass bulb) is embedded in the anthropomorphic breast phantom, in the lower outer quadrant of the left breast. The SPIO sample is removed from the breast phantom at approximately data point 855. The detector is moved by hand to and away from the sample insertion location ("surgical excision site").

S3. Video of experiment displayed in Fig. 9, showing the video recording alongside the screen recording of real-time data acquisition. An SPIO sample (500 ng Fe in a 3 mm-diam. glass bulb) is embedded in the anthropomorphic breast phantom, in the upper inner quadrant of the right breast. The SPIO sample is removed from the breast phantom at approximately data point 1016. The detector is moved by hand to and away from the sample insertion location ("surgical excision site").
